# The Mesothelioma epidemic in Western Europe: an update

**DOI:** 10.1038/sj.bjc.6601638

**Published:** 2004-03-02

**Authors:** C Pelucchi, M Malvezzi, C La Vecchia, F Levi, A Decarli, E Negri

**Affiliations:** 1 Istituto di Ricerche Farmacologiche ‘Mario Negri’, Via Eritrea 62, Milano 20157, Italy; 2 Istituto di Statistica Medica e Biometria, Università degli Studi di Milano, Milano 20133, Italy; 3 Institut Universitaire de Médecine Sociale et Préventive, Bugnon 17, Lausanne 1005, Switzerland; 4 Dipartimento di Scienze Biomediche e Biotecnologie, Sezione di Statistica Medica Università degli Studi di Brescia, Brescia 25123, Italy

**Keywords:** pleural cancer, mesothelioma, mortality trends, Western Europe

## Abstract

The number of male deaths from pleural cancer in France, Germany and Italy increased from about 8750 in 1990–1994 to 9550 in 1995–1999, suggesting that mesothelioma deaths in males may be levelling off in most of Western Europe.

Mortality from pleural mesothelioma is essentially a function of past exposure to asbestos, which is considered to be an early-stage carcinogen for mesothelioma (Peto *et al*, 1982). We applied a log-linear age, period and cohort model to pleural cancer mortality data to project the probable future trends in mesothelioma in Europe (La Vecchia *et al*, 2000). This model indicated a peak in the epidemic towards the end of the second decade of the current century (2015–2019) in most major Western European countries, and we estimated that about 250 000 male deaths from pleural cancer in the whole of Western Europe were in the period 1995–2029 (Peto *et al*, 1999).

However, a recent report from Sweden found that pleural mesothelioma incidence was levelling off in that country. Considering the timing and effect of the introduction of a ban on asbestos use, and the mesothelioma lag time following the exposure to asbestos, it was hypothesised that incidence peaks in other Western European countries might be anticipated between 2003 and 2013 (Hemminki and Li, 2003), slightly earlier than we had predicted. The authors stressed that their data should be considered cautiously, since the latency time from asbestos exposure to disease occurrence has been estimated as longer than 30 years (Bianchi *et al*, 1997), while asbestos imports to Sweden decreased markedly only in 1976 (Hemminki and Li, 2003). Similarly, a cohort analysis of Norwegian data showed a levelling of rates for men born after 1935 (Ulvestad *et al*, 2003), while an analysis of pleural mesothelioma incidence trends revealed a deceleration of the increase in some European countries (Montanaro *et al*, 2003).

Mortality data for the period 1995–1999, now available for most major European countries (WHO Mortality Database, 2003), have allowed us to compare observed and previously predicted deaths to assess the reliability of the age-cohort model previously used (Peto *et al*, 1999).

## MATERIALS AND METHODS

Methods for predicting the number of male deaths from mesothelioma have been reported elsewhere (Peto *et al*, 1999). Briefly, the age and birth cohort effects were estimated by fitting a log-linear Poisson model to the age-specific death rates, computed using data from the World Health Organisation (WHO), for each 5-year calendar period from 1970–1974 to 1985–1989.

From this WHO database, updated at January 2003, we have extracted, for Germany, France and Italy, the official number of male deaths from pleural cancer for the period 1990–1999. Pleural cancer was coded by two revisions of the International Classification of Diseases (ICDs) (163.0 under ICD-8, WHO, 1967 and 163 under ICD-9, WHO, 1977). We have excluded Switzerland and the Netherlands from this analysis, since these countries adopted, in 1995 and 1996 respectively, the Tenth Revision of the ICD that has introduced a specific code – C45 – for mesothelioma (WHO, 1992). Consequently, these data are not comparable with previous ones. Since Germany adopted ICD-10 in 1998, we considered mortality data for 1990–1994 and for 1997 only, that is, the central year of the period 1995–1999, instead of 1995–1997 to avoid any potential unbalancing upon the first half of the period. In any case, the number of male pleural mesothelioma deaths in Germany reported for 1995 (632 deaths) and 1996 (625 deaths) were closely comparable to 1997 (632 deaths).

Important changes occurred at this time in death registrations in Britain. In England and Wales, systematic medical enquiries to clarify ambiguous causes of death were stopped in 1993 (Office for National Statistics, 1996). Also, there were changes in the automatic cause coding. Consequently, British data are difficult to interpret and are not included in the present analysis.

To compute age-standardised mortality rates (ASMR), we used the direct method based on 5-year age groups and adjusted to the world standard population.

## RESULTS

For the period 1995–1999, the observed male deaths for all three countries considered were lower than predicted (Table 1

**Table 1:** Observed average number of male deaths per year from pleural cancer, their differences as compared to the predicted value (%) and ASMRs per 100 000 men, in three Western European countries in 1990–1999

	**40–54 years**		**55–64 years**		**65–84 years**		**Total**		
	**Observed**	**% difference *vs* predicted**	**Observed**	**% difference *vs* predicted**	**Observed**	**% difference *vs* predicted**	**Observed**	**% difference *vs* predicted**	**ASMR**
France 1990–1994	66	−8.3%	137	−8.1%	354	−6.3%	557	−6.9%	1.42
France 1995–1999	63	−23.2%	140	−16.7%	455	−7.7%	658	−11.4%	1.54

Germany 1990–1994^a^	102	—	183	—	339	—	624	−0.2%	1.13
Germany 1995–1999^b^	63	−12.5%	214	−4.5%	355	1.1%	632	−2.3%	1.05

Italy 1990–1994	77	1.3%	138	−1.4%	351	3.2%	566	2.0%	1.30
Italy 1995–1999	60	−16.7%	156	−9.8%	402	−3.4%	618	−6.4%	1.31

a Data on the age groups were not available.

b Observed data are for 1997 only.

). Observed numbers were 2.3% lower than predicted for Germany, 6.4% for Italy and 11.4% for France. For all countries, the difference between the observed and predicted values was largest in the youngest age group and smallest in the oldest.

The number of male pleural cancer deaths was similar in Germany in 1997 as in 1990–1994. The observed values were lower in 1995–1999 than in 1990–1994 in all countries at ages 40–54 years. In contrast, the observed number of deaths were all higher in 1995–1999 in the 65–84 years age group. The highest age-standardised mortality rate was reported for France (1.54), followed by Italy (1.31) and Germany (1.05). In 1997, for the first time since 1970–1974, the increasing trend in the age-standardised male death rates reversed in Germany. The Italian age-standardised death rate increased from 1.14 in 1985–1989 to 1.30 in 1990–1994, and then remained stable. France showed the highest mortality rate, still increasing in 1995–1999 (+8% compared with 1990–1994, +126% compared with 1970–1974).

## DISCUSSION

The total number of male deaths from pleural mesothelioma in France, Germany and Italy has continued to increase during the last decade of the 20th century, growing from approximately 7550 in 1985–1989 to 8750 in 1990–1994, and 9550 in 1995–1999. However, for the latest period, all the observed values were lower than predicted (Peto *et al*, 1999). This, together with the stabilisation of ASMRs and the consistent decrease in mortality at younger ages, supports the hypothesis that the rising trend in deaths from mesothelioma in men should level off in the near future in the above three countries as in Sweden (Hemminki and Li, 2003).

It appears therefore that our models have overestimated the number of mesothelioma deaths in men. For this, there are several possible explanation. First, the data for the more recent cohorts and those potentially involved in the first decrease of rates were based on only a few observations (the 1945 birth cohort was based on two rates) and on few deaths, so that there was a wide random variation. Further, we assumed that the 1950 birth cohort experienced the same rates as the 1945 cohort (which had the highest rates), and that the 1955 birth cohort would have had 50% lower rates (Peto *et al*, 1999). But apparently, the rates in the 1945 birth cohort were at the start of a decreasing trend, although it is unlikely that this by itself can explain the overestimation since the observed values were also generally lower than those predicted for the older age groups.

Thus, to explain the development of the mesothelioma epidemic, other factors need to be invoked, such as the type of asbestos mainly used in different countries over time (Hodgson and Darnton, 2000), duration of exposure and years since last exposure (Hauptmann *et al*, 2002). It does, however, appear that the number of asbestos-related mesothelioma deaths during 1995–2029 in Europe is likely to be lower than the 250 000 previously estimated on data available up to 1994.
